# Single-Molecule Sequencing (PacBio) of the *Staphylococcus capitis* NRCS-A Clone Reveals the Basis of Multidrug Resistance and Adaptation to the Neonatal Intensive Care Unit Environment

**DOI:** 10.3389/fmicb.2016.01991

**Published:** 2016-12-15

**Authors:** Patrícia Martins Simões, Hajar Lemriss, Yann Dumont, Sanâa Lemriss, Jean-Philippe Rasigade, Sophie Assant-Trouillet, Azeddine Ibrahimi, Saâd El Kabbaj, Marine Butin, Frédéric Laurent

**Affiliations:** ^1^Department of Clinical Microbiology, Northern Hospital Group, Hospices Civils de LyonLyon, France; ^2^International Centre for Research in Infectious Diseases, Institut National de la Santé et de la Recherche Médicale U1111, University of LyonLyon, France; ^3^National Reference Center for Staphylococci, Hospices Civils de LyonLyon, France; ^4^Biotechnology Laboratory (Medbiotech), Medical and Pharmacy School, University Mohammed V de RabatRabat, Morocco; ^5^Department of Clinical Microbiology, Eastern Hospital Group, Hospices Civils de LyonLyon, France; ^6^Department of Biosecurity PCL3, Laboratory of Research and Medical Analysis of the Fraternal of Gendarmerie RoyaleRabat, Morocco; ^7^Neonatal Intensive Care Unit, Eastern Hospital Group, Hospices Civils de LyonLyon, France

**Keywords:** bacteremia, multiple drug resistance, late-onset sepsis, SMRT, nisin, comparative genomics, *Staphylococcus capitis*

## Abstract

The multi-resistant *Staphylococcus capitis* clone NRCS-A has recently been described as a major pathogen causing nosocomial, late-onset sepsis (LOS) in preterm neonates worldwide. NRCS-A representatives exhibit an atypical antibiotic resistance profile. Here, the complete closed genome (chromosomal and plasmid sequences) of NRCS-A prototype strain CR01 and the draft genomes of three other clinical NRCS-A strains from Australia, Belgium and the United Kingdom are annotated and compared to available non-NRCS-A *S. capitis* genomes. Our goal was to delineate the uniqueness of the NRCS-A clone with respect to antibiotic resistance, virulence factors and mobile genetic elements. We identified 6 antimicrobial resistance genes, all carried by mobile genetic elements. Previously described virulence genes present in the NRCS-A genomes are shared with the six non-NRCS-A *S. capitis* genomes. Overall, 63 genes are specific to the NRCS-A lineage, including 28 genes located in the methicillin-resistance cassette SCC*mec*. Among the 35 remaining genes, 25 are of unknown function, and 9 correspond to an additional type I restriction modification system (*n* = 3), a cytosine methylation operon (*n* = 2), and a cluster of genes related to the biosynthesis of teichoic acids (*n* = 4). Interestingly, a tenth gene corresponds to a resistance determinant for nisin (*nsr* gene), a bacteriocin secreted by potential NRCS-A strain niche competitors in the gut microbiota. The genomic characteristics presented here emphasize the contribution of mobile genetic elements to the emergence of multidrug resistance in the *S. capitis* NRCS-A clone. No NRCS-A-specific known virulence determinant was detected, which does not support a role for virulence as a driving force of NRCS-A emergence in NICUs worldwide. However, the presence of a nisin resistance determinant on the NRCS-A chromosome, but not in other *S. capitis* strains and most coagulase-negative representatives, might confer a competitive advantage to NRCS-A strains during the early steps of gut colonization in neonates. This suggests that the striking adaptation of NRCS-A to the NICU environment might be related to its specific antimicrobial resistance and also to a possible enhanced ability to challenge competing bacteria in its ecological niche.

## Introduction

Coagulase-negative staphylococci (CoNS) are common commensals of the human skin and mucosa and are also opportunistic pathogens responsible for infections associated with indwelling medical devices and bloodstream infections (Otto, 2009). Among CoNSs, *Staphylococcus epidermidis* is the most prevalent species and is classically involved in nosocomial bacteremia in patients with co-morbidities including very low-birth weight preterm infants (Otto, 2009). However, the *Staphylococcus capitis* has also been incriminated in sepsis throughout the world in neonatal intensive care units (NICUs) (Van Der Zwet et al., 2002; Venkatesh et al., 2006; D'mello et al., 2008; Rasigade et al., 2012; Boghossian et al., 2013; Cui et al., 2013). We recently reported that a group of closely related multidrug-resistant (MDR) *S. capitis* strains belonging to the same clone, named NRCS-A, were present in most French NICUs but absent in the other pediatric or adult ICUs (Rasigade et al., 2012). In subsequent work, we demonstrated the dissemination of NRCS-A strains in distant NICUs throughout the world (Butin et al., 2016). This dissemination is of major concern because of the extensive drug resistance of NRCS-A as well as its ability to become highly endemic in some NICUs, representing up to 40% of all bacteremia cases (Stoll et al., 2002, 2005; Rasigade et al., 2012). Due to the presence of the type V-related staphylococcal chromosome cassette *mec* (SCC*mec*), all *S. capitis* NRCS-A strains are resistant to beta-lactams and exhibit reduced susceptibility to other antimicrobial agents in common use in NICUs, including resistance to aminoglycosides and resistance or heteroresistance to vancomycin (Venkatesh et al., 2006; Rasigade et al., 2012). Overall, the worldwide diffusion of a multidrug-resistant *S. capitis* clone that is highly adapted to NICU antibiotic selective pressure raises questions about the potential genetic support of its epidemiological success.

Few genome sequences of *S. capitis* are publicly available to date. In addition, only a single study using the genome of a *S. capitis* isolate collected from a bloodstream infection in an adult has reported the genomic comparison of virulence factors between *S. capitis* and *S. epidermidis* (Cameron et al., 2015). Considering the specific importance NRCS-A in the NICU setting and its international dissemination, we present here the first closed genome sequence of the French NRCS-A prototype strain CR01, a comparison of the whole-genome sequences (WGS) of three other NRCS-A strains collected from distant countries (Australia, Belgium, the United Kingdom), and a description of the genomic features of this NRCS-A clone.

## Methods

### Genome sequencing and assembly

The four strains of *S. capitis*, CR01, CR03, CR04, and CR05, used in this study were part of a previously published collection, with all strains being of the international *S. capitis* NRCS-A clone (Butin et al., 2016). The strains were isolated from blood cultures of preterm infants diagnosed with neonatal sepsis in Neonatal Intensive Care Units (NICUs) from four different countries: France (isolate CR01), Belgium (isolate CR03), Australia (isolate CR04) and the United Kingdom (isolate CR05) (for further details see Supplementary Data).

Initially, whole-genome pyrosequencing (454 Life Sciences/Roche) was performed for all 4 isolates, as previously reported (Lemriss et al., 2014, 2015). Single-molecule real-time (SMRT) sequencing was then performed for strain CR01 to obtain a closed reference genome for this clone (Supplementary Data).

Sequencing for *de novo* assembly was performed using PacBio RS II (Menlo Park, CA, USA). High molecular weight DNA was sheared in a Covaris g-TUBE (Covaris, Woburn, MA, USA) to obtain 20 kb fragments. After shearing, the DNA size distribution was checked using a Fragment Analyzer (Advanced Analytical Technologies, Ames, IA, USA), and 5 μg of the sheared DNA was used to prepare a SMRTbell library with PacBio SMRTbell Template Prep Kit 1 (Pacific Biosciences, Menlo Park, CA, USA) according to the manufacturer's recommendations. The resulting library was size selected using a BluePippin system (Sage Science, Inc. Beverly, MA, USA) for molecules larger than 11 kb. The recovered library was sequenced using 1 SMRT cell with P6-C4 chemistry and MagBeads with a PacBio RSII system (Pacific Biosciences, Menlo Park, CA, USA) at 240 min movie length.

Based on the SMRT cell sequencing, we generated 50,876 post-filter polymerase reads with an average read length of 15.6 kb and an N50 read length of 21.1 kb. The average coverage was 265X; for *de novo* assembly, the PacBio module “RS_HGAP_Assembly.2” in SMRTpipe version v2.3.0 was used for continuous long reads (CLR) after polishing and error correction with Quiver, as described previously (Chin et al., 2013). DNA methylation was determined using the RS_Modification_and_Motif_Analysis protocol within SMRT Portal v2.30, with a standardized *in silico* false positive error of ~1%. Only motifs with a mean modification quality value (QV) >50 and a mean coverage of >100X were validated as being modified (https://github.com/PacificBiosciences/Bioinformatics-Training/wiki/Methylome-Analysis-Technical-Note). Two polished contigs, one corresponding to the chromosome and the other to the plasmid, were obtained. Circularization of both contigs was achieved by manual comparison and removal of regions of overlaps.

### Computational analysis

Automatic syntactic and functional annotation of the closed, reference genome of strain CR01 [EMBL accession number: LN866849 (chromosome) and LN866850 (plasmid)] was performed using the MicroScope platform pipeline (Vallenet et al., 2006, 2013). The annotations of the draft genomes of strains CR03 (EMBL accession number: CVUF01000001-CVUF01000031), CR04 (EMBL accession number: CTEM01000001-CTEM01000038) and CR05 (EMBL accession number: CTEO01000001-CTEO01000039) were also performed using the MicroScope platform pipeline, as published previously (Lemriss et al., 2014). Visual inspection and manual curation were carried out using the MaGe platform and BLAST searches against NCBI databases.

Virulence factors were identified using the Virulence Factors database (VFDB; http://www.mgc.ac.cn/VFs/, Chen et al., 2012) and tblastn (using the ncbi-blast-2.2.27+ suite, Camacho et al., 2009) searches against in-house databases of known virulence genes of staphylococci.

Prophage regions were identified using the PHAST server (http://phast.wishartlab.com, Zhou et al., 2011). The predicted results were confirmed using the MaGe platform.

For comparison, we obtained publicly available assemblies for six additional whole-genome sequences of *S. capitis*: strains QN1 (NCBI Ref. Seq.: NZ_AJTH00000000.1), VCU116 (NCBI Ref. Seq.: NZ_AFTX00000000.1), SK14 (NCBI Ref. Seq.: NZ_ACFR00000000.1), LNZR-1 (NCBI Ref. Seq.: NZ_JGYJ00000000.1), C87 (NCBI Ref. Seq.: NZ_ACRH00000000.1), and AYP1020 (NCBI Ref. Seq.: NZ_CP007601.1). All six genomes were aligned against the closed genome of isolate CR01 using Mauve Progressive v.2.3.1 (Darling et al., 2010) with default settings.

Resistance genes were identified by a combination of ResFinder v.2.1 (Kleinheinz et al., 2014) and tblastn searches using the complete, curated Antibiotic Resistance Proteins database (multifasta file) available at the Comprehensive Antibiotic Resistance Database (CARD) (McArthur et al., 2013). For ResFinder, cutoffs of 80% minimum length and 90% identity were used to search for resistance genes in CR03, CR04, and CR05 assemblies previously performed against the reference CR01 using Mauve Progressive to determine their location and genomic context. For detection of resistance genes using the CARD complete, curated Antibiotic Resistance Proteins database, tblastn searches against a database composed of the four genomes of strains CR01, CR03, CR04, and CR05 were performed.

Genes specific to the NRCS-A clone were identified using the MaGe platform “Gene Phyloprofiler” tool (Vallenet et al., 2013) and the four annotated genomes of NRCS-A and public *S. capitis* genomes. Briefly, genomes were compared in terms of gene content using pre-computed homologies and synteny groups. Pairwise comparisons between predicted protein sequences of the studied genome and the proteins of another genome allowed computation of ranked hits and determination of bidirectional best hits (BBH) (for each protein, the three best hits were retained). Putative orthologous relationships between two genomes were defined as gene couples satisfying the BBH criterion and an alignment threshold for homology set as a minimum of 50% sequence identity along 80% of the length of the smallest protein. Mauve alignments were also used to complement the genomic context, followed by manual curation of the putative genes exclusively found in only the four NRCS-A genomes.

Insertion sequences (IS) in the genomes were identified using the ISfinder analysis tool (Siguier et al., 2006). Both blastn and blastp queries were performed with default settings. Matches with an *e*-value smaller than 0.5 were manually curated. To confirm whether a given IS position was conserved in the other genomes analyzed, the closed genome of isolate CR01 was aligned with the three other NRCS-A genomes and with the six public genomes using Mauve Progressive v.2.3.1 (Darling et al., 2010) with default settings. ORFs within immediate proximity of conserved IS loci in the NRCS-A genomes were compared with the *S. capitis* reference genome to check for potential alterations in coding sequences, using both Mauve Progressive and Mage platform. genomic islands (GIs) were predicted using IslandViewer 3 (Dhillon et al., 2015) and confirmed by alignment with the three NCRS-A genomes and the six public *S. capitis* genomes using Mauve Progressive. IS insertions within GIs were predicted as mentioned above. NRCS-A-specific GIs were searched in the NCBI database using megablast (Camacho et al., 2009) query, limiting to the *Staphylococcus* taxon (taxid: 1279). Published GIs from reference genomes were searched against *Staphylococcus aureus* Mu50 (NCBI Reference Sequence: NC_002758.2), COL (NCBI Reference Sequence: NC_002951.2), MW2 (NCBI Reference Sequence: NC_003923.1), N315 (NCBI Reference Sequence: NC_002745.2), FPR3757 (NCBI Reference Sequence: NC_007793.1) and S0385 (NCBI Reference Sequence: NC_017333.1), and *S. epidermidis* RP62A (NCBI Reference Sequence: NC_002976.3) using the Mage platform [15]. Syntonomes of more than 3 ORFs and with identity greater than 33% were considered to be GIs.

### Investigation of *nsr* gene presence in clinical bacterial strains

A panel of 15 strains (Table 1) comprising clinical strains of *S. capitis* (NRCS-A and non-NRCS-A) were used for nisin susceptibility testing. All staphylococcal species were originally isolated from blood, and species assignment was determined using Matrix-Assisted Laser Desorption/Ionization Time of Flight Mass Spectrometry (MALDI-TOF Vitek MS, Biomerieux, France). After isolation, the strains were cultured on Horse Blood Agar (Biomerieux) and then stored at −80°. Testing for susceptibility to nisin was performed using the disk diffusion test adapted from EUCAST^1^. Briefly, nisin solutions were prepared using 2.5% nisin powder (Sigma Aldrich, USA) according to a previously described protocol (Piper et al., 2009). Sterile paper disks (Thermofisher Diagnostics, France) were soaked with 30 μg of nisin solution. After an overnight incubation at 36°C on Horse blood Agar (Biomerieux, France), a 0.5 McF was seeded on Mueller Hinton agar (MHE) plates (Biomerieux, France). A disk containing 30 μg of nisin was placed in the middle of the plate. The diameter of the inhibition zones was measured after overnight incubation of the inoculated MHE plates at 36°C. The average diameter of inhibition and SEM were determined from four independent experiments.

**Table 1:** **Bacterial strains used in the nisin susceptibility test**.

**Species**	**NRCS-A clone**	**Strain**	**Setting**	**Country**	***nsr* gene**
SC	yes	CR01	NICU	FR	+
SC	yes	CR03	NICU	BE	+
SC	yes	CR04	NICU	AUS	+
SC	yes	CR05	NICU	UK	+
SC	yes	CR07	NICU	FR	+
SC	yes	BC69	NICU	South K	+
SC	yes	AQ62	NICU	NO	+
SC	yes	AW77	NICU	NZ	+
SC	yes	BI76	NICU	USA	+
SC	no	AB51	ICU	AUS	−
SC	no	CR02	ICU	FR	−
SC	no	AR22	ICU	DK	−
SC	no	AY18	ICU	GR	−
SC	no	AZ72	ICU	Sing	−
SC	no	BA24	ICU	USA	−

*SC, Staphylococcus capitis; FR, France; BE, Belgium; UK, United Kingdom; AUS, Australia; South K, South Korea; NO, Norway; NZ, New Zealand; USA, United States of America; DK, Denmark; GR, Greece; Sing, Singapore; NICU, neonatal intensive care unit; ICU, intensive care unit (adults)*.

PCRs for detecting the *nsr* gene were performed using consensus primers: pF–word-5′ GGAGATATGGGACCTATGATTGCA 3′ and pR–word-5′ GCTGTAAkTTCwCCkGAACTkGC 3′. These primers were designed based on alignment of the *nsr* gene sequence found in strain CR01 (*S. capitis* clone NRCS-A) and sequences retrieved from NCBI after a blastn (ref) search for homologous sequences in staphylococcal WGS genomes [*S. hyicus* ATCC 11249 (acc. num. CP008747), *Staphylococcus* sp. TE8 (acc. num. NZ_JMGB00000000.1), *S. epidermidis* MC28 (acc. num. NZ_ATCZ00000000.2), *S. epidermidis* VCU128 (acc. num. NZ_AHLI00000000.1)]. PCR amplification was conducted in a final volume of 25 μL, with 2.0 μL bacterial DNA (QuickExtract kit, Qiagen), 2.5 μL 10x Reaction Buffer, 0.75 μL 50 mM MgCl2, 1 μL 10 mM forward primer, 1 μL 100 mM reverse primer, 4 μL 5 mM dNTPs, 1 μL each primer and 0.125 μL Taq polymerase (Eurobio, France). The cycling parameters were as follows: (i) initial denaturation for 3 min at 95°C; (ii) 34 cycles, with 1 cycle consisting of 30 s at 95°C, 60 s at 60°C, and 90 s at 72°C; and (iii) a final extension for 10 min at 72°C.

## Results

WGSs of four strains belonging to *S. capitis* clone NRCS-A and originating from NICUs in four distant countries were obtained to investigate lineage-specific virulomes, resistomes, and mobilomes as well as the presence of unshared genes using 454 pyrosequencing technology, as reported previously (Lemriss et al., 2014, 2015). Depending on the isolate, assembly of the draft genomes produced 26 to 39 contigs. Re-sequencing of strain CR01 using SMRT technology (PacBio) allowed for the generation of closed complete sequences for the chromosome and the single plasmid that was subsequently used to produce scaffolds for the three remaining WGSs.

The length of CR01's chromosome is 2,522,871 bp and exhibits a low G + C content (33.02%), as is expected for staphylococci. We identified and annotated 2419 protein-coding regions, 19 rRNAs, 62 tRNAs, 34 ncRNAs (including RNAIII), and 20 transposases associated with insertion sequence elements and transposons. Of note, gaps (identified in the draft genome assemblies of the four NRCS-A isolates (based on 454 pyro-sequencing)) frequently occur within the vicinity of transposases, suggesting that some of the 454 short read assemblies were unable to bridge the gaps associated with repetitive genomic elements. These data clearly emphasize the suitability of third-generation sequencing technologies, such as PacBio SMRT, for obtaining fully defined *de novo* assemblies and, thus, for overcoming the issue of both local and global repeats (Koren and Phillippy, 2015). The genomic characteristics of the NRCS-A isolates tested in this study are summarized in Table 2.

**Table 2:** **General genomic features of *S. capitis* strain CR01 compared with another three WGS NRCS-A genomes**.

**Features**	***S. capitis* strain CR01 (Fr)**	***S. capitis* strain CR03 (Be)**	***S. capitis* strain CR04 (Aus)**	***S. capitis* strain CR05 (UK)**
GC% content	33.02	32.81	32.80	32.84
Nb of phages	1	1	1	3
Nb of plasmids	1	1^a^	1^a^	0
Nb and type of insertion sequence (nb on chromosome + nb on plasmid)	IS256: 10 + 0 IS1272: 3 + 1 IS431mec-like: 2 + 3	IS256: 1^b^+ 0 IS1272: 1 + 0 IS431mec-like: 2 + 4	IS256: 1^b^+ 0 IS1272: 1 + 0 IS431mec-like: 2 + 0	IS256: 1^b^ IS1272: 0 IS431mec-like: 2

a *Putative plasmid, no definitive data about circularization*.

b *Insertion sequence isolated in a small contig, indicating probable multiple insertions in the complete genome*.

### Virulome

The presence of virulence-associated genes in the closed genome of strain CR01 and subsequently the draft genomes of strains CR03 (Be), CR04 (Aus), and CR05 (UK) were inferred by comparison with known virulence factors previously reported for *S. aureus, S. epidermidis*, and *S. haemolyticus* using the VFDB database (Chen et al., 2012). The findings were augmented with BLAST searches of well-characterized staphylococcal virulence factors and key regulators (Table 3). Comparison of the four NRCS-A genomes with the six published genomes of non-NRCS-A *S. capitis*, including QN1, VCU116, SK14, LNZR-1, C87, and AYP1020 strains, showed that none of the known staphylococcal virulence genes are exclusively carried by the NRCS-A clone.

**Table 3:** **Comparison of virulence factors in *S. capitis* NRCS-A, non-NRCS-A, and *S. epidermidis* after exclusion of virulence factors present only in *S. aureus***.

**Function**	**Gene**	***S. epidermidis***		***S. capitis* NRCS-A clone**				***S. capitis* non-NRCS-A**				
		**ATCC 12228**	**RP62A**	**CR01 (Fr)**	**CR03 (Be)**	**CR04 (Aus)**	**CR05 (UK)**	**SK14**	**VCU116**	**C87**	**QN1**	**LNZR-1**
Adherence	*atl*	+	+	+	+	+	+	+	+	+	+	+
	*ebh*	+	+	+	+	+	+	+	+	+	+	+
	*ebp*	+	+	+	+	+	+	+	+	+	+	+
	*icaR*	0	+	+	+	+	+	+	+	+	+	+
	*icaA*	0	+	+	+	+	+	+	+	+	+	+
	*icaD*	0	+	+	+	+	+	+	+	+	+	+
	*icaB*	0	+	+	+	+	+	+	+	+	+	+
	*icaC*	0	+	+	+	+	+	+	+	+	+	+
	*sdrF*	+	+	0	0	0	0	0	0	0	0	0
	*sdrG*	+	+	0	0	0	0	0	0	0	0	0
	*sdrH*	+	+	+	+	+	+	+	+	+	+	+
Exozymes	*sspB*	+	+	0	0	0	0	0	0	0	0	0
	*sspC*	0	+	0	0	0	0	0	0	0	0	0
	*lip*	+	+	+	+	+	+	+	+	+	+	+
	*geh*	+	+	+	+	+	+	+	+	+	+	+
	*sspA*	+	+	0	0	0	0	0	0	0	0	0
	*nuc*	+	+	+	+	+	+	+	+	+	+	+
Host immune invasion	*capABC*	+	+	+	+	+	+	+	+	+	+	+
Toxins	*hlb*	+	+	+	+	+	+	+	+	+	+	+
	*hld*	+	+	+	+	+	+	+	+	+	+	+
	*hemolysin*	0	+	+	+	+	+	+	+	+	+	+
	*hlIII*	+	+	+	+	+	+	+	+	+	+	+
Proteases	*ClpP*	+	+	+	+	+	+	+	+	+	+	+
	*ClpB*	+	+	+	+	+	+	+	+	+	+	+
	*ClpC*	+	+	+	+	+	+	+	+	+	+	+
	*ClpX*	+	+	+	+	+	+	+	+	+	+	+
Phenol-soluble modulins	*psm α*	+	+	+	+	+	+	+	+	+	+	+
	*psm β1a*	+	+	0	0	0	0	0	0	0	0	0
	*psm β1B*	+	+	+	+	+	+	+	+	+	+	+
	*psm β2*	+	+	+	+	+	+	+	+	+	+	+
	*psm β3*	+	+	0	0	0	0	0	0	0	0	0
	*psm δ*	+	+	+	+	+	+	+	+	+	+	+
	*psmε*	+	+	+	+	+	+	+	+	+	+	+
	*psm-mec*	+	+	0	0	0	0	0	0	0	0	0
Esterases	*esterase1*	+	+	+	+	+	+	+	+	+	+	+
	*esterase2*	+	+	+	+	+	+	+	+	+	+	+
HTH transcription factors	*SarA*	+	+	+	+	+	+	+	+	+	+	+
	*SarR*	+	+	+	+	+	+	+	+	+	+	+
	*SarV*	+	+	+	+	+	+	+	+	+	+	+
	*SarX*	+	+	+	+	+	+	+	+	+	+	+
	*SarZ*	+	+	+	+	+	+	+	+	+	+	+
	*MgrA*	+	+	+	+	+	+	+	+	+	+	+
	*Rot*	+	+	+	+	+	+	+	+	+	+	+

*The presence of virulence genes is indicated by the “+” sign*.

Interestingly, the virulome of the NRCS-A *S. capitis* genome was quite similar to that of *S. epidermidis* RP62A, including the *ica*ABDCR and *cap*ABC biofilm-related operons, Clp proteases and multiple copies of PSM beta type 1b in tandem (75% aa identity to PSMbeta1b of strain RP62) and one PSM alpha (59% aa identity) (Otto, 2012). All PSMs were found in the same genetic environment as in the *S. epidermidis* RP62A genome, with conservation of upstream and downstream genes.

As previously observed for the fully closed *S. epidermidis* RP62a and *S. epidermidis* ATCC 12228 genomes (Zhang et al., 2003; Gill et al., 2005), no *S. aureus* toxins, except beta- and delta-hemolysins, were identified in any of the 10 *S. capitis* genomes (four NRCS-A and six public non-NRCS-A). Similarly, none of the genes associated with secretion systems (*esx*A, *esx*B, *esa*A, *esa*B, *esa*C, *ess*A, *ess*B, and *ess*C) or *S. aureus* serine proteases (*spl*A, B, C, D, E, and F) are present in the *S. capitis* genomes.

### Resistome

Several antibiotic resistance-associated genes were identified and correlated with the specific resistance phenotype previously reported for the NRCS-A clone (Rasigade et al., 2012; Table 4). All are located on mobile genetic elements (MGEs). Resistance to aminoglycosides is related to the bifunctional aminoglycoside-modifying gene *aacA-aphD*, which is carried on the transposon *Tn*4001 (GenBank accession no. AB682805.1; Lyon et al., 1984). Resistance to methicillin is due to the presence of a composite SCC*mec*-SCC*cad*/*ars*/*cop* cassette exclusively present in the NRCS-A clone, as recently published by our team (Martins Simões et al., 2013). Genomic comparison between the four NRCS-A strains showed that this mobile element is nearly identical: all ORFs are conserved (100% aa homology), and the few nucleotide sequence variations occur only in the CRISPR repeat region, as is expected for these regions. As previously reported (Martins Simões et al., 2013), no other antibiotic resistance genes were detected for this composite SCC element. Finally, the plasmidic *bla* operon, coding for *bla*Z beta-lactamase and its regulators, is present in all NRCS-A strains, except strain CR05 (UK).

**Table 4:** **Genomic profiles of antibiotic resistance for clone NRCS-A strains from four different countries**.

**Antibiotic**	**Gene**	**Strains**				**Genomic context**
		**CR01 (FR)**	**CR03 (Be)**	**CR04 (Aus)**	**CR05 (UK)**	
Penicillin	*blaZ*	+	+	+	−	Plasmid
Methicillin	*mecA*	+	+	+	+	Chromosome
Tetracycline	*tetK*	−	−	+	−	Plasmid^*^
Aminoglycosides	*aac(6′)-aph(2″)*	+	+	+	+	Chromosome
Macrolide, Lincosamide and Streptogramin B	*msr(A)*	−	−	+	−	Plasmid^*^
Fusidic acid	*far/fusB*	−	−	−	+	Phage (chromosome)^*^

All antibiotic resistance genes were found in MGEs. Putative regions in the draft genomes are indicated by an ^*^.

With regard to other antimicrobial families, for which variability in resistance profiles have been observed among NRCS-A isolates, phenotypic antimicrobial susceptibility testing matched the specific resistance gene contents of each NRCS-A strain. Plasmidic *msr*A and *tet*K genes, respectively involved in erythromycin resistance (with a negative D-test) and tetracycline resistance, were only identified in strain CR04 (Australia). In strain CR05 (UK), the gene *far*1, which is responsible for fusidic acid resistance, is carried by a putative phage.

### Mobile elements

**Figure 1:**
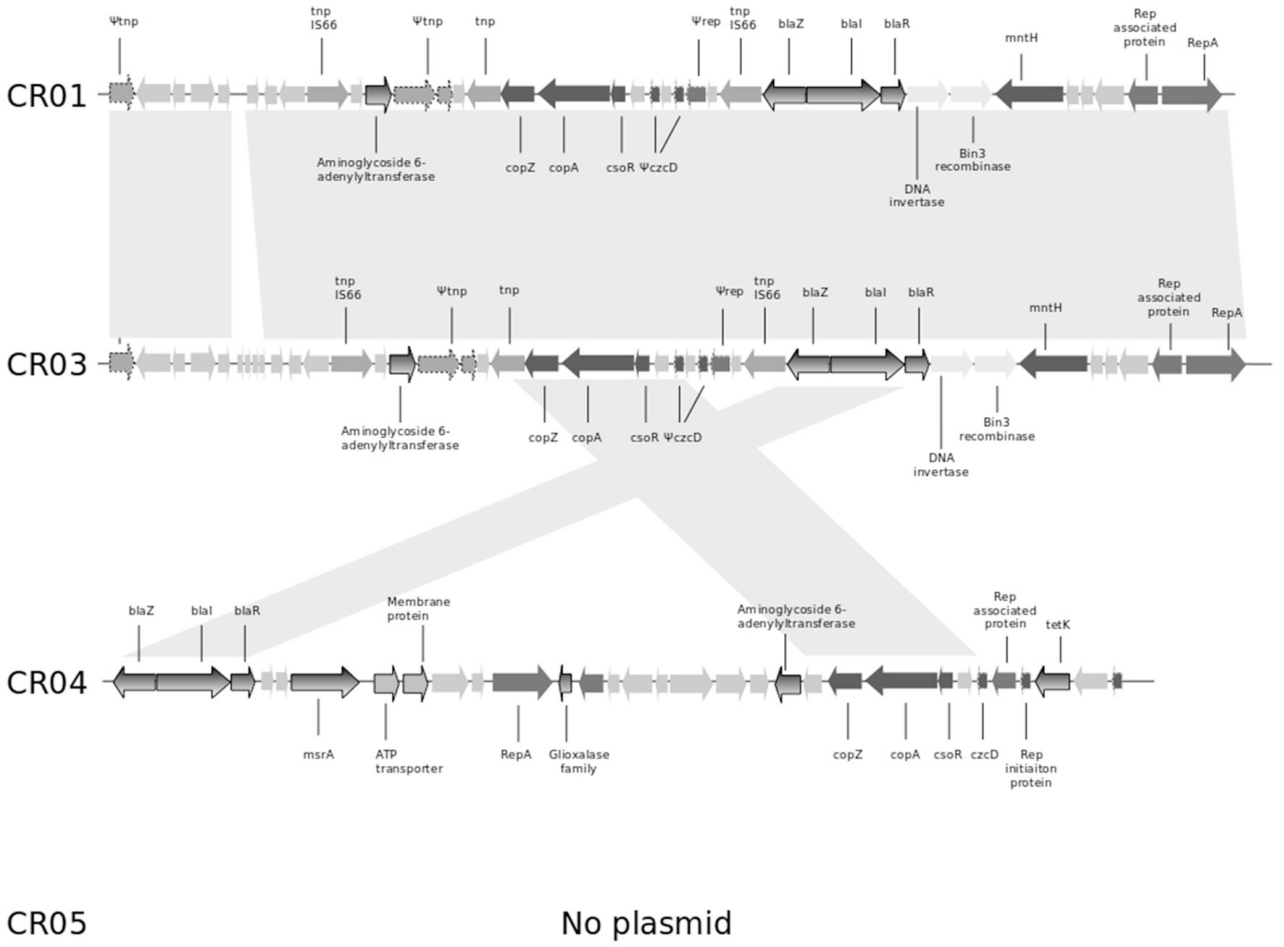
**Comparison of the genetic content of the strain CR01 plasmid with the draft genomes of the other three NRCS-A strains**. Open reading frames (ORFs) are shown as arrows indicating the direction of transcription. Homologous gene clusters are indicated by a light gray shadow connecting ORFs present in distinct plasmid sequences. Light gray arrows represent unknown proteins, unless specified otherwise. Arrows with dotted lines represent partial or truncated ORFs. Antibiotic resistance genes are colored with a gray gradient; genes associated with resistance to heavy metals are colored in dark gray. Abbreviations: *tnp*, transposase; *cop*Z, copper insertion chaperone and transporter component; *cop*A, copper transporter ATPase; *cso*R, copper-sensing transcriptional repressor; *czc*D, potassium/proton-divalent cation antiporter; *rep*, replication-associated family protein; *rep*A, replication-associated protein RepA; *bla*Z, beta-lactamase resistance gene; *bla*R, regulatory protein BlaR1; *bla*I, penicillinase repressor; *mnt*H, divalent metal cation transporter MntH, *tet*K, tetracycline resistance gene.

As mentioned previously, the shortest circular sequence obtained by SMRT for strain CR01 corresponds to a plasmid of 26.140 bp (GC content of 29.25%). It harbors 35 ORFs, including (i) the *bla* operon coding for penicillinase resistance to penicillins (see above) and (ii) copper resistance-related genes such as *cop*Z, *cop*A, and *cso*R (copper transcriptional repressor; Figure 1). Genome comparison of strain CR01's plasmid with the three other NRCS-A draft genomes revealed that the plasmid is not conserved in all NRCS-A strains (Figure 1). In strain CR03 (Belgium), one contig presents 31 of the 35 ORFs identified on the CR01 plasmid. Conversely, in strain CR04 (Australia), only 9 of the 35 ORFs (corresponding to the *blaZRI* operon and copper resistance operon) were detected in a contig corresponding to a putative plasmid. Finally, no putative plasmid was detected in strain CR05 (UK).

**Figure 2:**
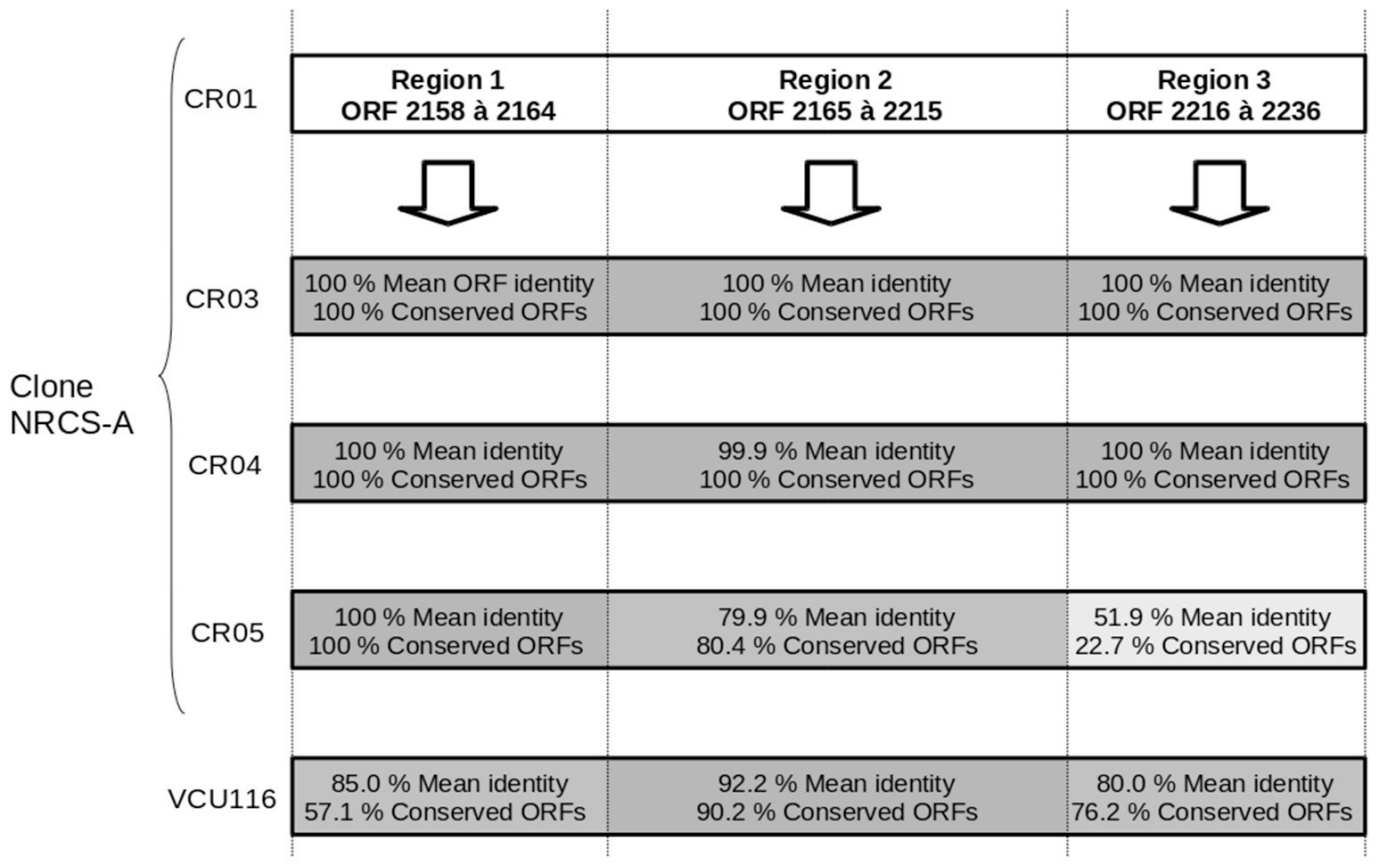
**Comparison of the genetic content of the strain CR01 intact prophage present in the other three draft genomes of NRCS-A strains**. Prophage prediction was performed using PHAST software; cR01's prophage is divided into three regions according to the degree of conservation compared to the other strains. The mean percent of aa identity in each region is indicated, and a gray gradient is also used to represent the percent similarity.

One intact prophage region was identified in all four NRCS-A genomes. This region shows 100% aa identity to the genomes of strains CR01, CR03, and CR04, but it is degenerate in CR05 (Figure 2). Moreover, this phage exhibits strong homology to a phage present in strain VCU116 [57.1, 90.2, 76.2% aa homology for regions 1, 2 and 3, respectively (Figure 2)] and less than 30% homology with phage Staphy_StB20_(NCBI Ref. Seq. NC_019915), the closest phage found in the NCBI database. Of note, two additional incomplete prophage regions were also detected in strain CR05.

Nineteen distinct insertion sequence (IS) elements, clustering into three distinct families (type IS256, *n* = 4; type IS1272, *n* = 5; IS431mec, *n* = 10), were identified in the CR01 genome. The presence of type IS256 was confirmed in all four NRCS-A strains, with two IS256 elements being associated with the aminoglycosides resistance gene aacA-aphD (′aac(6′)-aph(2″) on transposon *Tn*4001. Type IS1272 was also found in strains CR03 and CR04 but not in strain CR05, and their genomic locations are not conserved. Two IS431*mec* cassettes are present in all NRCS-A genomes, as they are carried within the SCC*mec*-SCC*cad*/*ars*/*cop* element that is specific to and conserved among the NRCS-A lineage (Martins Simões et al., 2013).

Finally, other GIs (*n* = 5) were predicted for CR01 (Table 2) and found in the CR03, CR04, and CR05 strains. However, only one was found exclusively in the NRCS-A lineage. This GI (bp position: 150,780–158,140 in the strain CR01 genome) contains six ORFs: one putative phosphoglycolate phosphatase, one conserved protein of unknown function and three pseudogenes of phage proteins (bp position: 151,470–152,238). Unexpectedly, two of these pseudogenes present 40 and 46.5% aa identity to *Listeria monocytogenes* phage B025 protein gp27 and the third pseudogene presents 60.6% aa identity to *L. monocytogenes* phage B025 protein gp28. Both gp27 and gp28 proteins have unknown functions.

### Methylome and restriction modification systems

SMRT technology (PacBio) allows for genome-wide detection of modified nucleotides based on the rate at which DNA polymerase incorporates bases during sequencing (Flusberg et al., 2010, Nat. Methods; Roberts et al., 2013). Analysis of polymerase kinetic profiles in strain CR01 identified 1333 methylated positions (Table S2) including 94% (*n* = 1253) corresponding to adenine methylations (m6A) and 0.006% (*n* = 8) to cytosine methylations (m4C). The adenine modifications observed correlate with the presence of two adenine restriction-modification systems (*hsd*MSR 1 pos: 476,473–482,360 bp; *hsd*MSR 2 pos: 686,750–691,866 bp) in the strain CR01 genome, whereas the presence of an *mcr*BC 5-methylcytosine restriction system (bp position 487,030–492,276) correlates with the cytosine modifications detected. Interestingly, the latter is associated in tandem with the specific additional *hds*MSR 1 operon located immediately after the SCC*mec*-SCC*cad*/*ars*/*cop* element (see above).

### NRCS-A clone specific genes

Comparison of the closed genome of strain CR01 with the draft genomes of the three other NRCS-A genomes and with the six public non-NRCS-A genomes revealed a unique set of 63 genes present exclusively in the NRCS-A clone genome (Table S1). Of these, 28 ORFS are carried by the composite SCC*mec*-SCC*cad*/*ars*/*cop* mobile element, which is related to the acquisition of methicillin resistance (*mec*A gene). Interestingly, within this cassette, the CRISPR element is specific to the NRCS-A lineage (Martins Simões et al., 2013). Of note, both the clone-specific cassette (28 genes) and its genetic environment (downstream and upstream regions) are fully conserved in the four NRCS-A strains, which confirmed that the four isolates belong to the same clonal population, even though they were isolated from distant countries/continents. Indeed, it is highly unlikely that the same SCC*mec* element (100% identity) could be acquired in four independent events in four distant countries.

Among the 35 remaining genes found exclusively in the NRCS-A lineage, 25 are of unknown function, and 10 correspond to the following: (i) an additional type I restriction modification system (*hsd*MSR, *n* = 3 genes), (ii) a cytosine methylation operon (*mcr*BC, *n* = 2), (iii) a cluster of four genes known to be involved in the biosynthesis of teichoic acids [*isp*D (2-C-methyl-D-erythritol 4-phosphate cytidylyltransferase), *tar*J (ribitol-5-phosphate dehydrogenase), *tar*K (Glycosyl glycerophosphate), and *tag*F (CDP-glycerol glycerophosphotransferase), *n* = 4]. Of note, the tenth gene encodes a 316 aa protein presenting 41% aa homology with the determinant for resistance to nisin (*nsr* gene) that is present in some *Lactococcus lactis* strains (Froseth and McKay, 1991; Liu et al., 2005) and which shows protease activity. Contrary to the plasmidic localization of the *nsr* gene in nisin-resistant *L. lactis* strains, the *nsr* gene in the NRCS-A clone is located on the chromosome (position: 521,747–522,694 bp) immediately upstream from the potassium (K+) transporter *kdp*EDABC operon conserved in all *S. capitis* isolates and downstream from the four teichoic acid biosynthesis genes (*isp*D, *tar*J, *tar*K, and *tag*F; see above). No IS or transposon-like region was identified near the *nsr* gene.

### Phenotypic assay of clone NRCS-A nisin resistance

To assess functional expression of the nisin resistance gene (nsr) found exclusively in the NRCS-A clone, we performed a disk diffusion test with nisin-charged disks using a collection of *S. capitis* strains, including nine strains belonging to the NRCS-A clone and six *nsr*-negative, non-NRCS-A strains. The *S. capitis* strains belonging to the NRCS-A clone presented significantly lower nisin inhibition zones (mean of four independent measurements ± SEM: 8.81 mm ± 1.31) than the strains isolated from adults that do not carry the *nsr* gene (16.5 ± 1.5; the Student *t*-test, *p* < 0.001 vs. 8.8 mm ± 1.3 in NRCS-A). This confirms that the *nsr* gene found exclusively in *S. capitis* strains belonging to the neonatal NRCS-A clone is functional and confers resistance to nisin. Nonetheless, it is known (i) that *S. aureus* (SA) can modulate its resistance to nisin and other small antibacterial peptides via two-component systems (TCSs) GraSR, NsaSR, BraSR in association with ABC transporters (Howden et al., 2010; Blake et al., 2011; Falord et al., 2011; Kolar et al., 2011; Kawada-Matsuo et al., 2013) and (ii) that SA strains resistant or heteroresistant to vancomycin (VISA and hVISA) usually present increased cell wall thickening (Cui et al., 2003). The same mechanisms have been observed in *L. lactis* resistant to nisin (Kramer et al., 2006; Shin et al., 2016). Thus, to assess whether the resistance to nisin observed in the NRCS-A strains is due exclusively to expression of the *nsr* gene and not to a cell wall-thickening adaptation, complementary experiments are required using *nsr*+*/nsr*− isogenic isolates. Such analyses are beyond the scope of the present paper.

## Discussion

The NRCS-A clone is of major interest due to its worldwide high dissemination in NICUs and its prevalence as an agent of neonatal sepsis in preterm newborns (Butin et al., 2016). Comparison between the complete genome of the prototype strain belonging to the *S. capitis* NRCS-A clone (strain CR01, France) with the draft genomes of clinical *S. capitis* isolates belonging or not to the NRCS-A clone revealed the presence of multiple MGEs mediating the atypical antibiotic resistance profile of this clone. These data confirm (i) the ability of the NRCS-A clone to adapt to the specific selective pressure of the antibiotics used in NICUs and (ii) emphasize the ability of WGS to provide accurate predictions of the resistance phenotypes of staphylococci and to become a promising alternative for culture methods.

Based on genomic comparisons using previously characterized staphylococcal virulence genes, the NRCS-A virulome is highly similar to that of other non-NRCS-A *S. capitis* as well as *S. epidermidis* RP62A strains, which suggests that the success of this clone in neonates is likely not due to increased virulence. Nonetheless, we identified the exclusive presence of a gene encoding a functional nisin resistance (*nsr*) gene in the NRCS-A lineage that is seldom found in staphylococci.

Nisin, a 34 aa antimicrobial peptide produced by a group of gram-positive *Lactococcus* and *Streptococcus* species (Shin et al., 2016), is active against a wide range of gram-positive bacteria, including staphylococci. Its mode of action is dual, involving (i) inhibition of cell wall biosynthesis (transglycosylation step), as it binds to and sequesters lipid II from its functional location, and (ii) pore formation (Wiedemann et al., 2001; Peschel and Sahl, 2006; Egan et al., 2016). Nisin has been described as a key player of the gut barrier, and it is widely used as food preservative due to its potent bactericidal activity. Moreover, it has been shown that (i) expression of the *nsr* gene, identified in *Streptococcus agalactiae*, in *L. lactis* not producing nisin induces resistance to nisin and (ii) the presence of human nisin-producing lactic acid bacteria in the gut reduces intestinal colonization by vancomycin-resistant enterococci (Millette et al., 2008).

Taken together, these data and our results strongly suggest that nisin resistance might be responsible for the potential increase in the ability of the NRCS-A clone to establish itself as part of the initial microflora of neonates, in which the *Lactococcus* genus is one of the dominant bacterial taxa (Park et al., 2005; Morelli, 2008). Expression of the NSR peptidase might enable NRCS-A isolates to establish themselves in the gut of neonates and also to colonize it and survive longer than nisin-susceptible bacteria. This may explain the over-representation of these isolates in sepsis processes, either through direct translocation in blood (Taft et al., 2015) or via colonization of indwelling devices. Although it remains unknown how *S. capitis* NRCS-A strains are able to translocate into the bloodstream of infants, it is hypothesized that the entry point might be the digestive tract, which is immature in very preterm infants (Taft et al., 2015). Further studies are needed to fully characterize the digestive microflora of very low-weight preterm-infants and its potential impact on the selection of and the fitness advantage to NRCS-A isolates.

## Author contributions

PM: work, study design, data analysis, and manuscript preparation. HL: data analysis and manuscript preparation. YD: data analysis, work, and manuscript preparation. SL: work and manuscript preparation. JR, SA, AI, and SE: manuscript preparation. MB and FL: study design and manuscript preparation.

## Funding

The present work was financed by the French Ministry of Health and the French Institute for Public Health Surveillance (INVS) - Santé publique France in the framework of the National Reference Center of Staphylococci and by the grant ING20111223510 from the Fondation pour la Recherche Medical (FRM).

### Conflict of interest statement

The authors declare that the research was conducted in the absence of any commercial or financial relationships that could be construed as a potential conflict of interest.

## Footnotes

^1^European Committee on antimicrobial Susceptibility Testing, 2015, http://www.eucast.org/fileadmin/src/media/PDFs/EUCAST_files/Disk_test_documents/Manual_v_5.0_EUCAST_Disk_Test.pdf
