# Supplementary material for: Single-Molecule Sequencing (PacBio) of the Staphylococcus capitis NRCS-A Clone Reveals the Basis of Multidrug Resistance and Adaptation to the Neonatal Intensive Care Unit Environment
Source: Front Microbiol. 2016 Dec 15;7:1991. doi: 10.3389/fmicb.2016.01991 (PMC5157051; doi:10.3389/fmicb.2016.01991)
Supplement: Supplementary file 1 [file DataSheet1.DOC]

Supplementary Data

**“Single Molecule sequencing of a *Staphylococcus capitis* from the NRCS-A clone reveals the basis of multidrug resistance and adaptation to Neonatal Intensive Care Units environments”**

P Martins Simões *et al.* 2015, Frontiers in Microbiology (submitted)

# **Bacterial isolates and Growth conditions**

1. All four strain of the S. capitis NRCS-A clone were isolated from blood cultures of preterm infants with presenting a Late-onset sepsis (LOS) and hospitalized in NICUs from 4 distinct countries (strain CR01, is from an in infant in an NICU in France, strain CR03 from an in a NICU in Belgium, strain CR04 from a NICU ion Australia and strain CR05 from an NICU in the United Kingdom).
2. Species identification of the bacterial isolates and antimicrobial susceptibility testing (AST) were performed, respectively, using Vitek MS (bioMérieux, Marcy l’Etoile), 16S rDNA sequencing, the automated BD Phoenix system (Becton Dickinson, Sparks, MD) and with Shimadzu-MALDI-TOF MS system (Shimadzu Corporation), as implemented on [1].
3. The strain was identified as being a S*taphylococcus capitis by* VITEK MS with 99.9% and at 93.7% by the MALDI-TOF MS, using the Shimadzu Launchpad software program and the SARAMIS database application (AnagnosTec GmbH) for automatic measurement and identification. The antimicrobial susceptibility test (AST) results were analyzed according to the recommendations of the French Microbiology Society [2]. The *S. capitis* bacteremia was considered positive based on a single positive blood culture [3,4]. All 4 isolates were resistant to penicillin, methicillin, gentamicin, rifampin, hetero-resistant to vancomycin and sensitive to fusidic acid and fluoroquinolones.

# **DNA isolation**

1. All samples were prepared for sequencing by growing the *S. capitis* CR01,CR03, CR04 and CR05 strains aerobically at 37°C in BloodAgar for 24-48 hours. Genomic DNA was then extracted using the PureLink^TM^ genomic DNA kit (Invitrogen^TM^) according to the manufacturer’s recommended protocol. DNA quantification was performed using a NanoVue ^TM^ Plus (HVD Life Sciences).
2. **Genome sequencing and assembly**
3. 454-shotgun libraries were constructed using 1 µg of DNA, for each sample, and following the GS Rapid library protocol (Roche 454, Roche) with a mean library size of 1547 bp (strain CR03), 1874 bp (strain CR04) and 1419 bp (strain CR05). The resulting 454 DNA libraries were sequenced using a high-throughput whole-genome shotgun strategy performed on a Genome Sequencer FLX+ system (454 Life Sciences/Roche) using FLX Titanium reagents, according to the manufacturer’s protocols and instructions. De novo assemblies were performed using the Roche Newbler (version 2.9) software package, and the sequencing results are summarized below.

**References:**

1. Gagnaire J, Dauwalder O, Boisset S, Khau D, Freydière A-M, Ader F, et al. (2012) Detection of Staphylococcus aureus Delta-Toxin Production by Whole-Cell MALDI-TOF Mass Spectrometry. PLoS ONE 7(7): e40660. doi:10.1371/journal.pone.0040660
2. French Society for Microbiology. Recommandations du Comite de l'Antibiogramme de la Societe Francaise de Microbiologie. 2009. Available at: [wwwsfmassofr/doc/downloadphp?doc=DiU8C&fic=casfm_2009pdf](http://wwwsfmassofr/doc/downloadphp?doc=DiU8C&fic=casfm_2009pdf). Accessed 22 February 2009.
3. Rasigade J-P, Raulin O, Picaud J-C, Tellini C, Bes M, Grando J, Ben Saïd M, Claris O, Etienne J, Tigaud S, Laurent F. Methicillin-Resistant Staphylococcus capitis with Reduced Vancomycin Susceptibility Causes Late-Onset Sepsis in Intensive Care Neonates. PLoS ONE 2012;7(2):e31548.
4. Hall KK, Lyman JA. Updated review of blood culture contamination. Clin Microbiol Rev. 2006 Oct;19(4):788-802.

**Table S1 – Genes found exclusively in the *S. capitis* NRCS-A clone and not in other *S. capitis* public genomes**

| **CR01 ORFs annotation** | **Gene** | **Product** | **Genomic context** |
| --- | --- | --- | --- |
| CR01_v3_0498 | hsdM | putative type I restriction enzyme HindVIIP M protein | Additional restriction modification system type I, immediately after the composite SCCmec-SCCcad/ars/cop element |
| CR01_v3_0499 | hsdS | HsdS homologue |
| CR01_v3_0500 | hdsR | putative type I restriction enzyme HindVIIP R protein |
| CR01_v3_0529 | nsr | Nisin-resistance protein | nisin resistance gene |
| CR01_v3_2157 | _ | protein of unknown function | Phage region |
| CR01_v3_2158 | _ | putative lipoprotein |
| CR01_v3_2159 | _ | conserved protein of unknown function |
| CR01_v3_2162 | _ | protein of unknown function |
| CR01_v3_0176 | _ | conserved protein of unknown function |  |
| CR01_v3_0520 | ispD | 2-C-methyl-D-erythritol 4-phosphate cytidylyltransferase | possibly a biosynthesic pathway |
| CR01_v3_0521 | tarJ | putative ribitol-5-phosphate dehydrogenase |
| CR01_v3_0522 | _ | conserved protein of unknown function |
| CR01_v3_0523 | _ | conserved protein of unknown function |
| CR01_v3_0429 | _ | conserved protein of unknown function | SCCmec element of the composite SCCmec-SCCcad/ars/cop element |
| CR01_v3_0430 | _ | conserved protein of unknown function |
| CR01_v3_0431 | _ | conserved protein of unknown function |
| CR01_v3_0432 | _ | conserved protein of unknown function |
| CR01_v3_0433 | _ | conserved protein of unknown function |
| CR01_v3_0434 | _ | putative primase |
| CR01_v3_0435 | _ | Cassette chromosome recombinase C7 |
| CR01_v3_0448 | _ | Transcriptional regulator, DeoR family |
| CR01_v3_0449 | _ | conserved protein of unknown function |
| CR01_v3_0450 | _ | conserved protein of unknown function |
| CR01_v3_0451 | _ | conserved protein of unknown function |
| CR01_v3_0452 | _ | conserved protein of unknown function |
| CR01_v3_0453 | ccrC | Cassette chromosome recombinase C |
| CR01_v3_0458 | _ | conserved protein of unknown function |
| CR01_v3_0459 | _ | truncated hsdR |
| CR01_v3_0460 | cas | CRISPR-associated endonuclease Cas1 |
| CR01_v3_0461 | cas | CRISPR-associated endoribonuclease Cas2 |
| CR01_v3_0462 | csm | CRISPR-associated Csm1 family protein |
| CR01_v3_0463 | csm | CRISPR-associated Csm2 protein |
| CR01_v3_0464 | csm | CRISPR type III-associated RAMP protein Csm3 |
| CR01_v3_0465 | csm | CRISPR-associated RAMP protein |
| CR01_v3_0466 | csm | CRISPR-associated RAMP protein, Csm5 family |
| CR01_v3_0467 | csm | CRISPR-associated protein, Csm6 family |
| CR01_v3_0468 | cas | CRISPR-associated endoribonuclease Cas6 |
| CR01_v3_0469 | _ | conserved protein of unknown function, potential pseudo? |
| CR01_v3_0470 | _ | conserved membrane protein of unknown function |
| CR01_v3_0471 | _ | Transcriptional regulator, TetR family |
| CR01_v3_0472 | _ | conserved membrane protein of unknown function |
| CR01_v3_0310 | _ | exported protein of unknown function |  |
| CR01_v3_0311 | _ | conserved protein of unknown function |  |
| CR01_v3_0312 | _ | conserved protein of unknown function |  |
| CR01_v3_0183 | _ | protein of unknown function |  |
| CR01_v3_0496 | _ | protein of unknown function |  |
| CR01_v3_0497 | _ | conserved protein of unknown function |  |
| CR01_v3_0505 | _ | conserved protein of unknown function |  |
| CR01_v3_0507 | _ | McrBC 5-methylcytosine restriction system component |  |
| CR01_v3_0508 | _ | protein of unknown function |  |
| CR01_v3_0178 | _ | protein of unknown function |  |
| CR01_v3_0179 | _ | protein of unknown function |  |
| CR01_v3_0180 | _ | protein of unknown function |  |
| CR01_v3_0673 | _ | protein of unknown function |  |
| CR01_v3_0674 | _ | conserved protein of unknown function |  |
| CR01_v3_0675 | _ | conserved protein of unknown function |  |
| CR01_v3_0676 | _ | protein of unknown function |  |
| CR01_v3_0677 | _ | protein of unknown function |  |
| CR01_v3_0680 | _ | conserved protein of unknown function |  |

**Table S2 – Methylated motifs detected for *S. capitis*  NRCS-A strain CR01**

All methylated motifs presented in this table were validated by checking that the mean modification QV was >50 and the mean motif coverage was >100.

| Motif | Modified Position | Type of modification | No. detected motifs^a^ | No motifs in genome | % of methylated/detected motifs | Type of RM system | Partner motif |
| --- | --- | --- | --- | --- | --- | --- | --- |
| TTAYNNNNGTC | 3 | m6A | 1253 | 1256 | 99.46 | I | GACNNNNRTAA |
| ANGCAGNTCTNNNNA | 4 | m4C | 8 | 10 | 80.0 | II |  |
| GCGGTANYANNB | 3 | unknown | 40 | 67 | 59.7 | ? |  |

^a^ The total number includes motifs occurring on the “+” and “-” strands ( partner motif is located in the “-” stra nd)
